# Activation metrics for structural connectivity recruitment in deep brain stimulation

**DOI:** 10.1093/braincomms/fcaf301

**Published:** 2025-08-19

**Authors:** Konstantin Butenko, Jan Roediger, Bassam Al-Fatly, Ningfei Li, Till A Dembek, Yifei Gan, Guan-Yu Zhu, Jianguo Zhang, Andrea A Kühn, Andreas Horn

**Affiliations:** Movement Disorders and Neuromodulation Unit, Department of Neurology with Experimental Neurology, Charité-Universitätsmedizin Berlin, Corporate Member of Freie Universität Berlin and Humboldt-Universität zu Berlin, Berlin 10117, Germany; Center for Brain Circuit Therapeutics, Department of Neurology, Brigham & Women's Hospital, Harvard Medical School, Boston, MA 02115, USA; Movement Disorders and Neuromodulation Unit, Department of Neurology with Experimental Neurology, Charité-Universitätsmedizin Berlin, Corporate Member of Freie Universität Berlin and Humboldt-Universität zu Berlin, Berlin 10117, Germany; Berlin Institute of Health at Charité—Universitätsmedizin Berlin, BIH Biomedical Innovation Academy, BIH Charité Junior Digital Clinician Scientist Program, Berlin 10117, Germany; Movement Disorders and Neuromodulation Unit, Department of Neurology with Experimental Neurology, Charité-Universitätsmedizin Berlin, Corporate Member of Freie Universität Berlin and Humboldt-Universität zu Berlin, Berlin 10117, Germany; Movement Disorders and Neuromodulation Unit, Department of Neurology with Experimental Neurology, Charité-Universitätsmedizin Berlin, Corporate Member of Freie Universität Berlin and Humboldt-Universität zu Berlin, Berlin 10117, Germany; Department of Neurology, Faculty of Medicine, University of Cologne, Cologne 50937, Germany; Department of Neurosurgery, Beijing Tiantan Hospital, Capital Medical University, Beijing 100070, China; Department of Neurosurgery, Beijing Tiantan Hospital, Capital Medical University, Beijing 100070, China; Department of Neurosurgery, Beijing Tiantan Hospital, Capital Medical University, Beijing 100070, China; Movement Disorders and Neuromodulation Unit, Department of Neurology with Experimental Neurology, Charité-Universitätsmedizin Berlin, Corporate Member of Freie Universität Berlin and Humboldt-Universität zu Berlin, Berlin 10117, Germany; Center for Brain Circuit Therapeutics, Department of Neurology, Brigham & Women's Hospital, Harvard Medical School, Boston, MA 02115, USA; MGH Neurosurgery & Center for Neurotechnology and Neurorecovery (CNTR) at MGH Neurology Massachusetts General Hospital, Harvard Medical School, Boston, MA 02114, USA; Network Stimulation Institute, Department of Stereotactic and Functional Neurosurgery, University Hospital Cologne, Cologne 50937, Germany

**Keywords:** deep brain stimulation, axonal activation, probabilistic modeling, electric field, pathway activation modeling

## Abstract

Comparatively high excitability of myelinated fibres suggests that they represent a major mediator of deep brain stimulation effects. Such effects can be modelled using different levels of abstraction, ranging from simple electric field estimates to complex multicompartment axon models. In this study, we explored three metrics to evaluate axonal activation: electric field magnitudes, electric field projections and pathway activation modelling. Furthermore, in order to account for variability in axonal morphology, these metrics were computed in a probabilistic fashion. To showcase and illustrate their relevance, we retrospectively analysed a dataset of 15 Parkinson’s disease patients, who were stimulated in the subthalamic nucleus in bipolar mode. High similarity of activation patterns was observed for the electric field metrics, but not for pathway activation modelling, which might be attributed to its ability to capture stimulation’s polarity. Nevertheless, all three metrics associated motor improvement with activation of motor pallidosubthalamic and hyperdirect pathways. To make these probabilistic approaches accessible to the community, the modelling and statistical framework was implemented in the openly available Lead-DBS toolbox.

## Teaser

Butenko *et al*. compared simple and advanced approximations of axonal activation considering the uncertainty in fibre diameters. For bipolar stimulation, the discrepancy in estimated activation depended on the fibre trajectory relative to the stimulation field. Moreover, the prominent effect of fibre diameter selection further confirmed the necessity of probabilistic approaches.

## Graphical Abstract

**Graphical Abstract:**
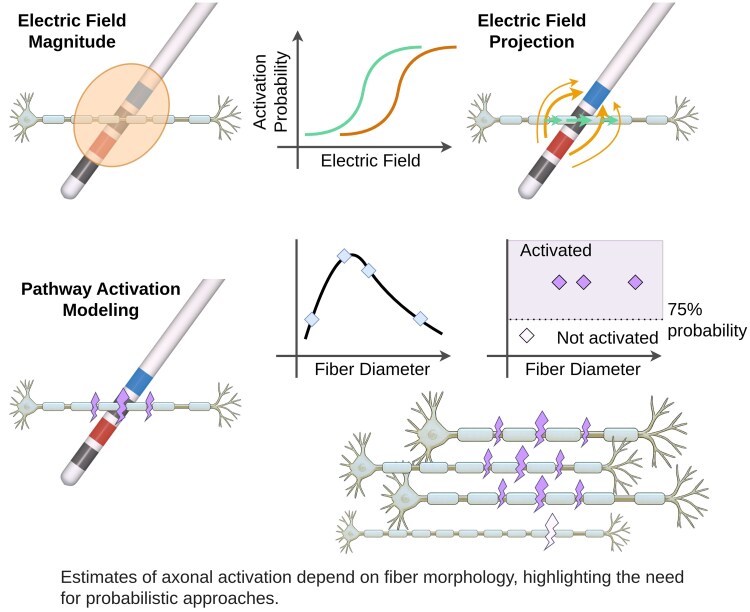

## Introduction

In deep brain stimulation (DBS), various modelling approaches have been considered to quantify effects on white matter tracts. Based on the Hodgkin–Huxley model,^1^ an activating function was proposed, defined by the second spatial derivative of the extracellular electric potential.^2^ To capture an accumulated effect of the stimulation along the fibre, driving force methods were proposed.^3,4^ For a single contact stimulation, however, activation may be approximated by the electric field magnitude.^5^ Increasingly, studies have binarized the magnitude (often at 200 V/m^6,7^) to delineate stimulation volumes, sometimes mistakenly referred to as the volume of tissue activated (VTA). In its original definition,^8^ VTA quantifies stimulus responses of axon models placed on a grid around the lead. In pathway activation modelling (PAM), such axons instead follow anatomically meaningful trajectories of white matter tracts.^9^

To choose from this repertoire of methods, researchers have to understand their relevance for the given study.^10^ Beyond biophysical plausibility, a key factor to consider is whether the model provides binary or continuous (probabilistic) estimates of activation. Either type of result will dictate statistical tests for a group-level analysis.

In this paper, we describe three probabilistic activation metrics: electric field magnitude, electric field projection along the fibre, and probabilistic pathway activation modelling (pPAM), which incorporates uncertainty of axon model parameters. To contextualize the relevance of these metrics, we compare them using clinical data of Parkinson’s disease patients implanted in the subthalamic nucleus (STN) and stimulated using bipolar mode, which represents a non-trivial modelling case.^11^ To facilitate future studies, these metrics are implemented in the open-source toolbox Lead-DBS.^12^

## Materials and methods

### Concepts

**Figure 1:**
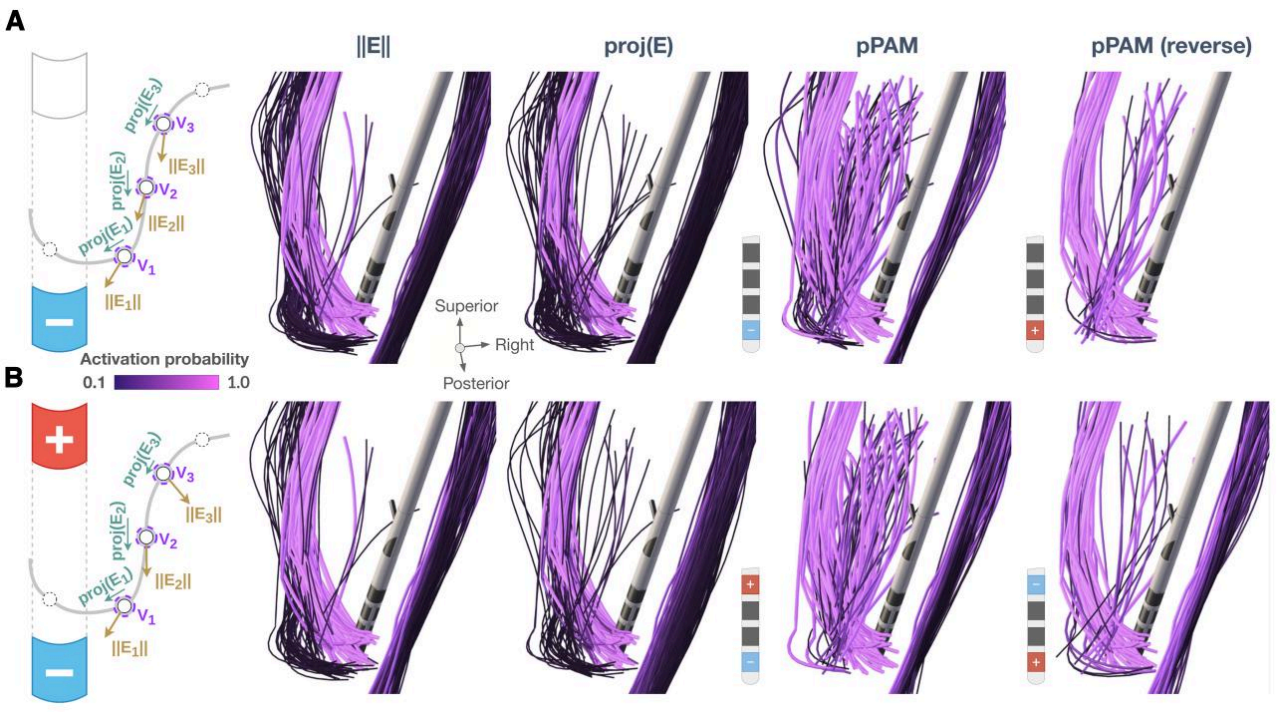
**Differences between the activation metrics for monopolar (A) and bipolar stimulations (B).** Left: In contrast to the electric field magnitude ||E||, the electric field projection proj(E) considers its relative direction to the fibre. The directionality is also encoded by the pathway activation model (PAM) through the stimulus-induced distribution of the extracellular potential (V) along the fibre. PAM relies on multiple biophysical parameters, e.g. myelination thickness and internodal spacing, which can be sampled probabilistically (pPAM). Right: Activation metrics computed for selected hyperdirect, pallidosubthalamic and corticospinal fibres.^18^ For sigmoid-transformed ||E|| and proj(E), the difference is observed for the pallidosubthalamic fibres, especially in the bipolar case. Anodic stimulation (estimated by pPAM) leads to lower activation of fibres passing or parallel to the lead (panel **A**, right). For bipolar stimulation, lower activation is predicted for short-passing fibres terminating closer to the anode (panel **B**, right).

DBS modelling provides a distribution of the electric vector field, and, subsequently, the electric field magnitude ||E||, used as a proxy for axonal activations.^5,11,13^ However, activation of a fibre is induced by the field component *parallel* to it.^14,15^ Thus, projecting the field onto it (the dot product of the field with the unit vector tangential to the fibre) yields a potentially more informative metric, see Fig. 1. Both the magnitude ||E|| and the projection *proj*(E) are evaluated at multiple points along the fibre, and a fibre-wise value can be defined as either a sum, mean or peak of these point-wise metrics. This fibre-wise value is then used as a regressor when analysing stimulated tissue and observed outcomes.^16,17^

However, neither ||E|| nor *proj*(E) encodes simultaneous depolarization and hyperpolarization effects of bipolar stimulations. For this, pathway activation modelling has to be applied. Beyond the electric field, PAM uses computational axon models, which require specification of multiple parameters, such as ion channel characteristics, myelin properties, internodal distances, etc. These parameters are subjected to uncertainties, and, therefore, we propose to model PAM in a probabilistic fashion. For example, fibre diameters may be sampled from distributions reported in the neuroanatomical literature. Once these parameters are sampled, the axon model is solved to estimate its response to the stimulation. If an action potential is detected, the axon is deemed ‘activated’.

Probabilistic PAM informs the certainty of activation that can be estimated as *N*_activated_ / *N*_samples_, where *N*_activated_—number of samples in which the axon is activated. For the electric field metrics, their values can be converted to activation probabilities using a sigmoidal function^19^: the higher the fibre-wise value, the higher the certainty of activation. While this approach does not explicitly model probabilistic parameters, it reflects that different axonal morphologies have different activation thresholds.^5^ The certainty may be leveraged in statistical analyses as a weight in a regression for continuous variables or in odds ratio statistics for binary variables.^19^

### Notes on implementation

The described methodology is implemented in Lead-DBS with the following steps. First, non-linear warps from the patient to standard space are obtained, and electrodes are localized.^12^ Second, a structural connectivity atlas is non-linearly warped into patient space. Then, electric field metrics and PAM are computed in patient space within OSS-DBS.^20^ PAM is repeated *N* times for *N* probabilistic samples using a NEURON^21^ implementation of McNeal’s axon model.^22^ For the statistical group-level analysis, the results are imported into the Fibre Filtering Explorer.^12^ This tool operates in the template space, but since the activation metrics are defined on the fibres (from the warped structural connectivity atlas), patient space results can be imported directly. This is a new feature of the toolbox, while previous studies in Lead-DBS evaluated activations in template space.

### Clinical data analysis

To explore these metrics on a group level, we retrospectively analysed 15 Parkinson’s patients with bilateral STN-DBS electrodes implanted at Charité—Universitätsmedizin Berlin and Beijing Tiantan Hospital. Patients were selected based on the availability of imaging data and clinical motor assessment, conducted at least three months after surgery, and only patients with bipolar stimulation settings were included. The collection and analysis of all patient data was approved by the Local Ethics Committee of Charité—Universitätsmedizin Berlin (master vote EA2/186/18) and the Local Ethics Committee of Beijing Tiantan Hospital (KY2022-006-02). All patients were implanted with omnidirectional four-level electrodes: 14—Medtronic (Minneapolis, MN, USA), 1—PINS (Beijing, China). For details on demographics, clinical outcomes and stimulation protocols, please refer to *Table 1*. Based on the pre- and post-operative MRI and post-operative CT imaging, DBS electrodes were localized following the standard pipeline implemented in Lead-DBS.^12^ This included computation of non-linear warps between the patient (native) space and template (MNI) space using Advanced Normalization Tools,^23^ a brain shift correction to account for possible pneumocephalus. To describe the structural connectivity around the STN region, the Basal Ganglia Pathway Atlas was employed.^1^ Electric fields and pathway activations were computed in a heterogeneous and dispersive volume conductor model using OSS-DBS software,^20^ and results were further processed as described in the previous section. To also model and compare *binary* activation metrics, electric field magnitudes were thresholded at the commonly used 200 V/m,^5^ while the threshold for electric field projections was adjusted to 125 V/m. This lower threshold represents a rough match for the projection metric, yielding a comparable amount of ‘activated’ fibres considered in the model.

**Table 1:** Patient demographics, stimulation settings and outcomes

ID	Age/Sex	Disease Duration (years)	UPDRS-III (med-off)		Lead Model	Stimulation Settings (Right/Left Hemisphere)			
			Stim-off	Stim-on		Contacts	Voltage (V)	Pulse Width (us)	Frequency (Hz)
1	65/M	14	36	16	M3389	0-1+/8-9+	4.5/3.5	90/90	130/130
2	60/M	10	55	36	M3389	1 + 2-/9-10+	5.0/4.6	90/90	70/70
3	51/F	9	38	18	M3389	2 + 3-/10-11+	1.8/2.2	60/60	130/130
4	46/M	7	70	26	M3389	1 + 2-3-/9 + 10-11+	4.7/4.1	80/80	95/95
5	45/M	n.a.	55	32	M3389	2 + 3-/10 + 11-	2.0/8.3	60/60	125/125
6	69/M	8	25	16	PINS L301	2 + 3-/7 + 6-	1.8/2.0	60/60	105/105
7	65/M	16	24	19	M3389	1 + 2-/8 + 9-	1.6/1.6	60/60	130/130
8	63/M	9	35	12	M3389	1 + 2/9 + 10-	1.6/0.5	60/60	130/130
9	62/M	6	29	25	M3389	1 + 2-/9 + 10-	2.8/2.6	80/80	160/160
10	51/M	7	41	23	M3389	0 + 1-/8 + 9-	1.9/1.9	60/60	130/130
11	68/F	3	60	33	M3387	1 + 2-/9 + 10-	2.0/2.8	60/80	130/130
12	62/F	9	25	8	M3389	2 + 3-/8 + 9-	2.5/2.2	70/70	130/130
13	60/M	14	26	22	M3389	2 + 3-/8 + 9-	2.0/2.7	60/70	145/145
14	71/F	10	33	22	M3389	2 + 3-/9 + 10-	2.3/2.3	70/70	90/90
15	66/M	7	15	9	M3389	0 + 1-/8 + 9-	2.3/2.3	70/60	130/130

M3387—Medtronic 3387; M3389—Medtronic 3389.

Probabilistic PAM was conducted for uniformly sampled fibre diameters (*N* = 10) in the range of 1–4 um defined based on the neuroanatomical literature.^24-26^ For binary PAM, two thresholding levels were used: with p(A) = 1.0 (the axon was activated for all fibre diameters) and p(A) ≥ 0.1 (the axon was activated for at least one fibre diameter). Axons intersecting with the electrode were flagged, and their activation was not probed. Instead, we computed the Euclidean distance from the centre point between the active contacts to each activated fibre. If the flagged fibre was closer to the centre point than the most remote activated fibre, then the flagged fibre was considered activated as well. The clinical effect of the structural connectivity recruitment was described by the per cent improvement of Unified Parkinson’s Disease Rating Scale III score (motor examination, UPDRS-III) between DBS-on and DBS-off conditions, both evaluated in the medication-off state during the same session.

### Statistical methods

To quantify differences and similarities of pathway activation profiles, i.e. per cent activations across pathways, paired sample t-tests and Spearman’s rank correlations were used. When analysing motor improvement, for each fibre, an unpaired two-sample *t*-test and its probability-weighted version were applied to compare outcomes across patients, in which the fibre was ‘activated’ and the ones in which it was not. Only fibres ‘activated’ (for probabilistic metrics p(A) ≥ 0.5) in at least 20% of stimulations were considered to avoid model overfitting. Assuming symmetry of DBS effects on motor symptoms, stimulations were ‘mirrored’ across the hemispheres when conducting the t-tests. No statistical significance is reported, and no multiple comparison correction was applied to these *t*-test results to avoid inflation of the activation profile discrepancy across different activation metrics and thresholding levels (smaller diameters correspond to lower activations and, consequently, smaller parameter spaces). Moreover, the significance of the computed *T*-values would have to be interpreted with caution, considering the relatively low number of analysed subjects (*N* = 15).

## Results

**Figure 2:**
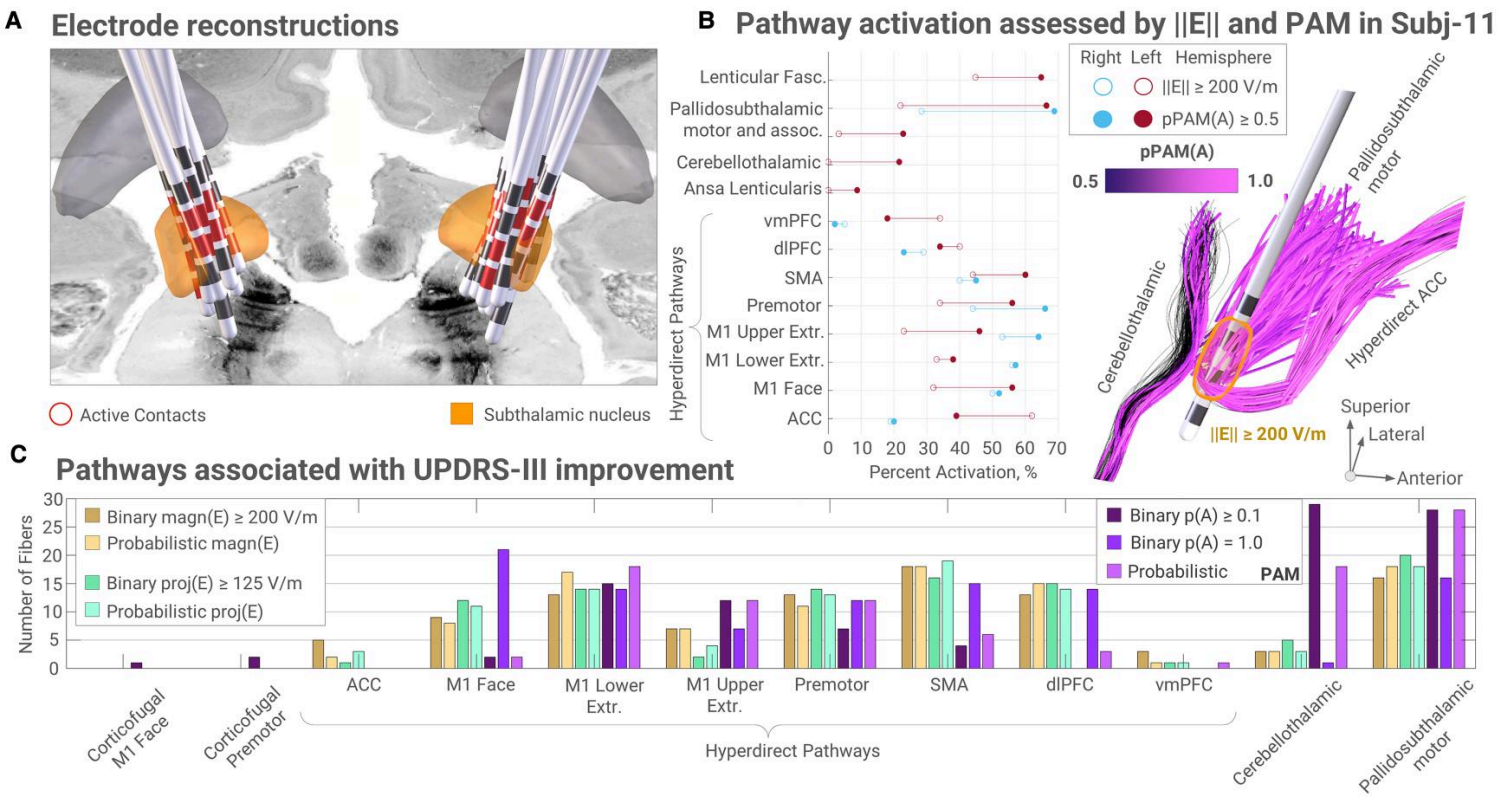
**Group-level analysis of 15 Parkinson’s patients with STN-DBS in bipolar mode. A:** Using Lead-DBS, electrode trajectories were reconstructed, showing localization to the subthalamic region. **B:** Detailed description of activation profiles computed using the thresholded electric field magnitude ||E|| (≥ 200 V/m) and binary pathway activation modelling (PAM) for subject 11. The right panel highlights a selection of left hemisphere pathways with a large activation discrepancy between the metrics. While ||E|| emphasizes orthogonally approaching trajectories, e.g. the hyperdirect projection from ACC, PAM estimates higher activation of fibres oriented more parallel to the electric field. **C:** Pathway-wise distribution of fibres associated with UPDRS-III improvement across the activation metrics. The distribution is computed based on 100 fibres with the highest *T*-values of fibre-wise unpaired two-sample *t*-tests (*N* = 30). Note the good agreement between the electric field metrics and the higher discrepancy for the PAM profiles. Cortical regions: M1—primary motor, ACC—anterior cingulate, dlPFC/vmPFC—dorsolateral and ventromedial prefrontal, SMA—supplementary motor area. Activation probabilities are referred to as pPAM(A) and p(A). Extr.—extremity, magn.—magnitude, proj.—projection, assoc.—associative, fasc.—fasciculus.

**Figure 3:**
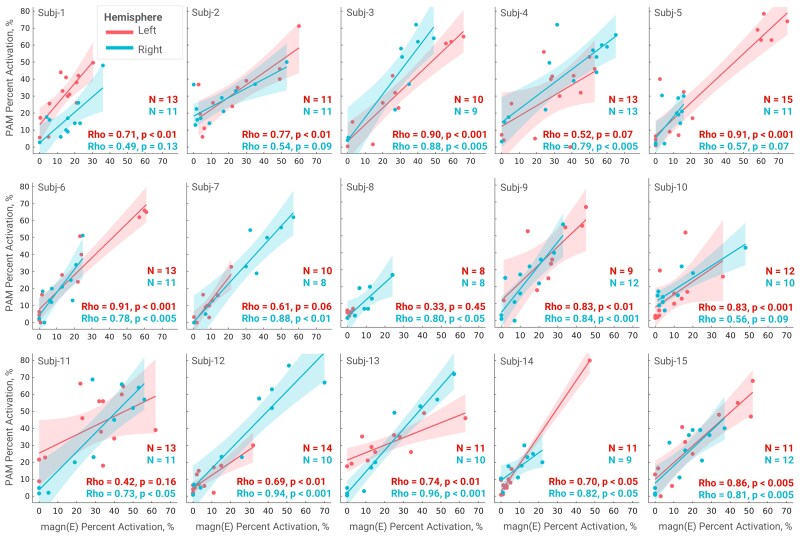
**Spearman’s rank correlations of pathway activation profiles computed based on ||E|| ≥ 200 V/m and binary PAM.** Each point denotes computed per cent activation for one pathway using both metrics. While PAM and ||E|| approaches showed comparable results in most cases, for 8 out of 30 electrodes, Spearman’s correlation did not reach statistical significance, which was partially attributed to the aforementioned orientation selectivity. Note that the correlation differs between the hemispheres due to differences in stimulation and electrode trajectory in relation to the fibres.

All electrodes were localized in the subthalamic region (Fig. 2*, panel A*). Using each metric, profiles of per cent activations of all pathways represented in the atlas were computed (Fig. S1*).* These profiles demonstrated varying degrees of agreement across patients and hemispheres, with Fig. 2*, panel B* showcasing an example where a large discrepancy in activation was observed for pathways of different orientations when estimated by ||E|| ≥ 200 V/m and PAM. Notably, this discrepancy was less prominent when comparing PAM with *proj*(E) for this case (see Fig. S2). Spearman’s rank correlations between activation profiles estimated by ||E|| ≥ 200 V/m and PAM ranged from 0.33 to 0.96 (mean Rho = 0.74) across all 30 hemispheres reaching statistical significance (*P* < 0.05) in 22 instances (see Fig. 3). *Proj*(E) and PAM showed a similar agreement (range, 0.32–0.96; mean Rho = 0.74, with statistical significance reached in 25 instances, see Fig. S2), and the paired sample *t*-test on the z-scored rank correlations showed non-significant difference between ||E|| and *proj*(E) correlations to the PAM profiles (*P* = 0.84). The ||E|| and *proj*(E) profiles were highly correlated (range, 0.63–1.0; mean Rho = 0.91, with statistical significance reached in 28 instances, see Fig. S3).

Statistical analyses of the pathways associated with UPDRS-III improvement also revealed differences across the metrics, see Fig. 2*, panel C*. The bar plot shows a distribution of 100 fibres with the highest T-values across the pathways. In contrast to the binary PAM, which assumed small fibre diameters (*P*(A) = 1.0), binary PAM for large diameters (*p*(A) ≥ 0.1) and the probability-weighted PAM emphasized the cerebellothalamic pathway, located posterior and dorsal to the STN. All metrics identified various branches of the motor hyperdirect pathway and the motor portion of the pallidosubthalamic tract. Note that the electric field metrics yielded more similar profiles, and a larger disagreement was observed for PAM assuming small fibre diameters (p(A) = 1.0).

## Discussion

In this study, we aimed at (i) introducing an open-source implementation of the structural connectivity activation metrics in the commonly used DBS analysis pipeline (ii) highlighting differences across these metrics in theoretical and clinically relevant cases. To this end, we systematically compared electric field magnitude, electric field projection, and pathway activation modelling—each implemented within a probabilistic framework—using an *in silico* example and in a cohort of Parkinson’s disease patients undergoing STN-DBS with bipolar stimulation.

The *in silico* example (Fig. 1) shows a prominent difference in pathway activations depending on the electric field directionality, which is ignored by the magnitude metric ||E||, but accounted for by the projection metric *proj*(E) and PAM. Notably, the difference is less evident for monopolar stimulations, where the electric field is more trivial. Another case is anodic stimulation that leads to a comparatively lower activation of passing^27^ and parallel fibres^28^ (Fig. 1*A, right panel*). When the polarity was flipped in the bipolar mode (the anode closer to the tracts, Fig. 1*B, right panel*), the activation remained similar for fibres traversing in the immediate vicinity of the lead and for longer fibres parallel to it, while activation of the shorter passing fibres was decreased. These results match clinical observations that higher anodic currents are typically required to achieve comparable clinical effects and side effects.^29,30^ Importantly, the change of polarity would not affect estimation of the electric field metrics, which has been recognized as their major limitation.^11^

To assess the clinical relevance of these considerations, we analysed 15 STN-DBS patients stimulated in bipolar mode. According to the fibre-wise distribution of T-values, all three metrics associated motor pallidosubthalamic and hyperdirect pathways with motor improvement. However, the observed differences between the metrics warrant discussion. The electric field magnitude and projection metrics showed high agreement, suggesting that selective pathway engagement via field directionality was limited in this cohort. This likely reflects the use of omnidirectional electrodes, which restrict current-steering capabilities. In contrast, PAM yielded distinctly different activation profiles, while the results of probabilistic PAM, which incorporated uncertainty in fibre diameters, indicated its prominent effect on the estimated activation. Further investigation into the axonal architecture could help to constrain this uncertainty and improve the accuracy of pathway-specific estimates.

## Conclusion

We illustrated and applied three probabilistic activation metrics for structural connectivity analysis in DBS. Projection of the electric field onto the fibre is theoretically more informative than the electric field magnitude. Practically, however, these metrics identified comparable structural connectivity profiles in the clinical dataset of bipolar stimulations with omnidirectional electrodes. In contrast, pathway activation modelling resulted in distinctly different activation patterns, which, nevertheless, also linked stimulation of the motor pallidosubthalamic and hyperdirect pathways with motor improvement. All metrics were implemented into one modeling and statistical framework and are openly available to facilitate research on novel stimulation paradigms.

## Supplementary Material

fcaf301_Supplementary_Data

## Data Availability

The project’s code is available at https://github.com/netstim/leaddbs/tree/develop. The used clinical data are available from the corresponding author upon reasonable request.
