# Supplementary material for: Activation metrics for structural connectivity recruitment in deep brain stimulation
Source: Brain Commun. 2025 Aug 19;7(5):fcaf301. doi: 10.1093/braincomms/fcaf301 (PMC12402687; doi:10.1093/braincomms/fcaf301)
Supplement: fcaf301_Supplementary_Data [file fcaf301_supplementary_data.pdf]

Supplementary Material for
"Activation Metrics for Structural Connectivity Recruitment in Deep Brain Stimulation"

Konstantin Butenko, Jan Roediger, Bassam Al-Fatly, Ningfei Li, Till A. Dembek, Yifei Gan, Guan-Yu Zhu, Jianguo Zhang, Andrea A. Kühn and Andreas Horn.

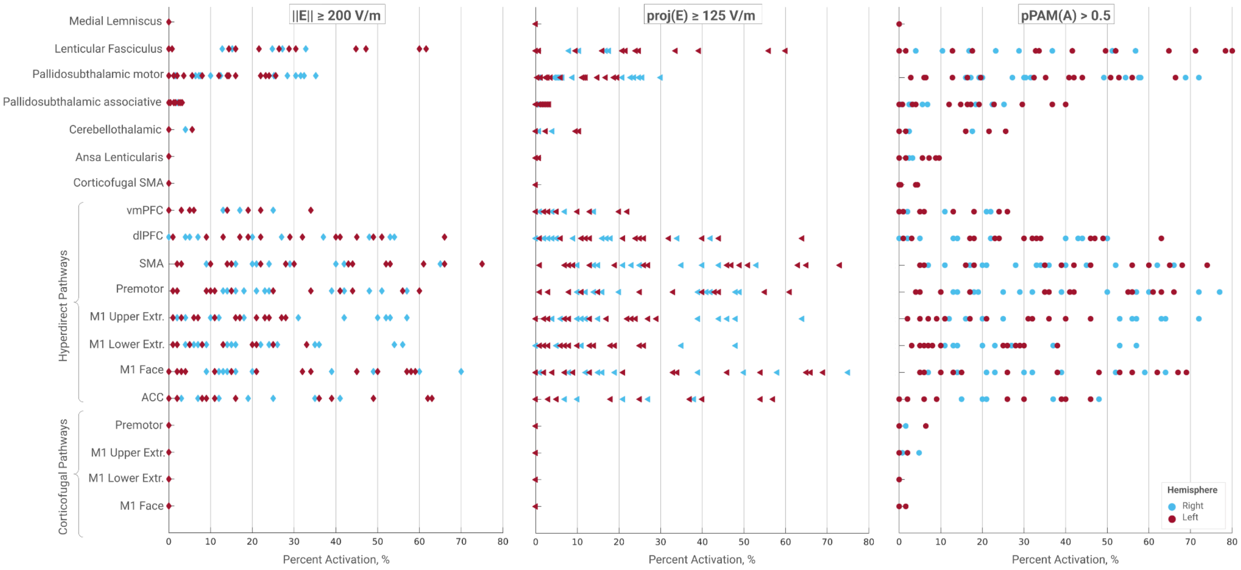

Figure S1: *Pathway percent activations of 15 STN-DBS patients quantified by three different metrics. While the activation profiles are generally comparable, especially for the electric field metrics (||E|| and proj(E)), a higher recruitment rate is estimated by pathway activation modeling (PAM) for some projections, e.g., pallidosubthalamic and cerebellothalamic pathways.*

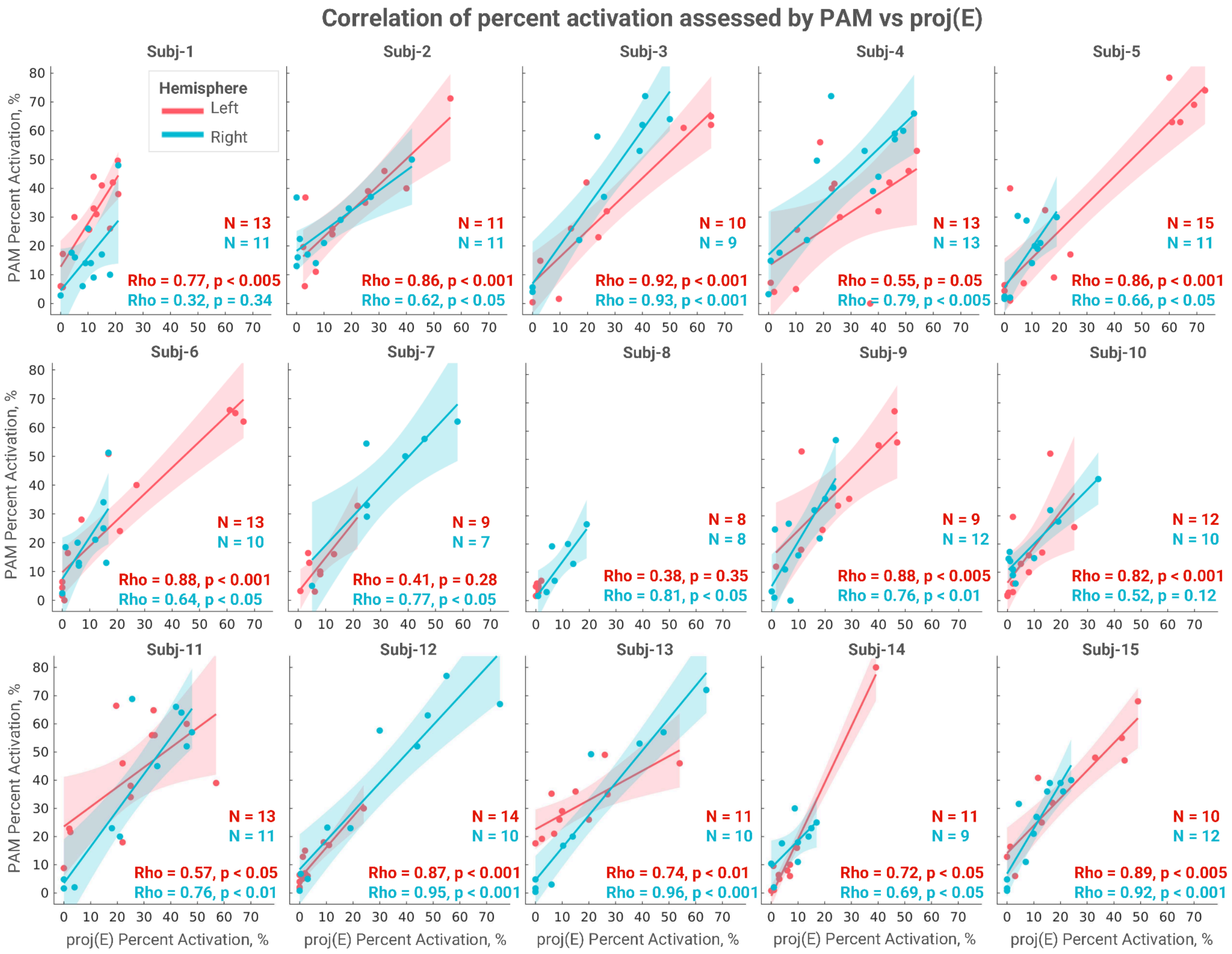

Figure S2: *Spearman's rank correlations of pathway activation profiles computed based on thresholded electric field projections (≥ 125 V/m) and binary pathway activation modeling. Each point denotes computed percent activation for one pathway using both metrics. For 5 out of 30 electrodes Spearman's correlation profiles did not reach statistical significance. Note that a paired sample t-test on the z-scored rank correlations did not reveal a significant difference with ||E|| correlations to the PAM profiles shown in Fig. 2, panel C (p = 0.84).*

Correlation of percent activation assessed by proj(E) vs ||E||

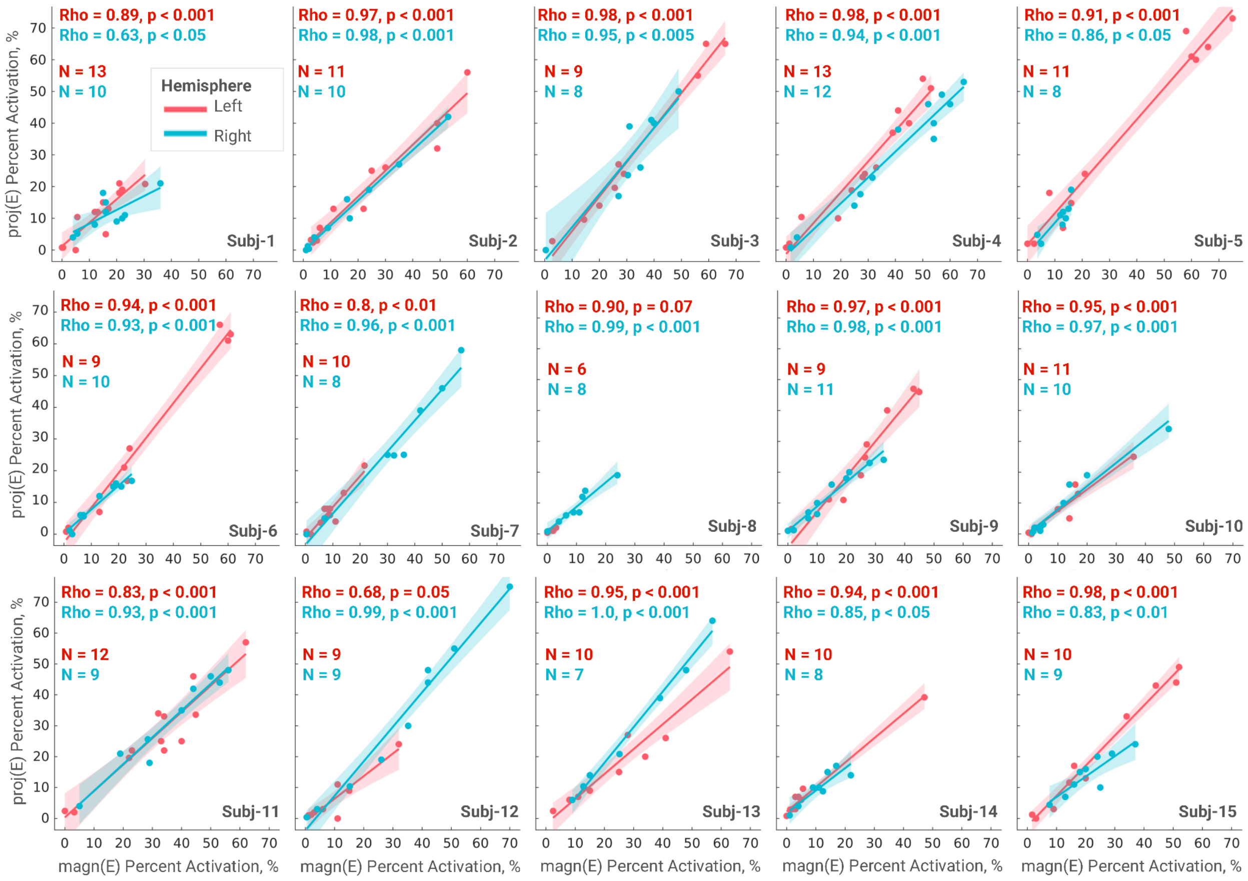

Figure S3: *Spearman's rank correlations of pathway activation profiles computed based on thresholded electric field magnitudes ||E|| (≥ 200 V/m) and projections (≥ 125 V/m). Each point denotes computed percent activation for one pathway using both metrics. The statistical significance was not reached in 2 out 30 cases.*
